# Biopolymer Honeycomb Microstructures: A Review

**DOI:** 10.3390/ma16020772

**Published:** 2023-01-12

**Authors:** Dominik Fajstavr, Klára Fajstavrová, Bára Frýdlová, Nikola Slepičková Kasálková, Václav Švorčík, Petr Slepička

**Affiliations:** Department of Solid State Engineering, University of Chemistry and Technology Prague, Technická 3, 166 28 Prague, Czech Republic

**Keywords:** honeycomb, polymer, biopolymer, replication, tissue engineering, antibacterial properties, surface modification, morphology, breath figure, improved phase separation

## Abstract

In this review, we present a comprehensive summary of the formation of honeycomb microstructures and their applications, which include tissue engineering, antibacterial materials, replication processes or sensors. The history of the honeycomb pattern, the first experiments, which mostly involved the breath figure procedure and the improved phase separation, the most recent approach to honeycomb pattern formation, are described in detail. Subsequent surface modifications of the pattern, which involve physical and chemical modifications and further enhancement of the surface properties, are also introduced. Different aspects influencing the polymer formation, such as the substrate influence, a particular polymer or solvent, which may significantly contribute to pattern formation, and thus influence the target structural properties, are also discussed.

## 1. Natural Honeycomb

Living organisms respond to environmental conditions in such a way as to best adapt their structure and functionality. Therefore, scientists have always considered nature to be the best source of inspiration for the development of new materials [1] and applications. One idea was to artificially imitate the perfect structure of honeycombs, which is, without doubt, one of the most fascinating formations in nature [2,3]. Natural honeycomb has an efficient internal structure, with thousands of hexagonal cells connected in highly ordered arrays. In the book of the Roman scholar M. T. Varro from 36 BC., we can find one of the first discoveries regarding the shape of beehive cells [4]. Varro wrote: “Does not the chamber inside the honeycomb have six angles... The geometers Euclid and Zenodorus proved that this hexagon inscribed in a circle encloses the largest part of space.” Subsequently, in the 4th century, the Greek mathematician Pappus wrote in the introduction to his book The Collection [5]: “By a certain geometrical foresight, the bees know that a hexagon is larger than a square or a triangle and will take more honey with the same consumption of material and labor.”

**Figure 1:**
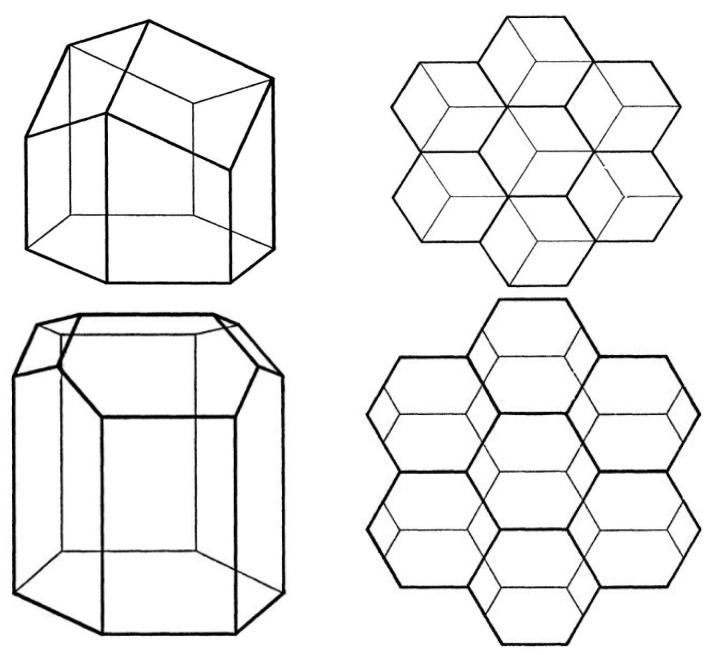
Honeycomb structures [1].

The spatial geometry of honeycomb, which was carefully observed by J. Kepler [6] and discussed by J. Hales [7], is shown in Figure 1. It is a hexagonal prism enclosed at one end by three rhombuses. Hexagonal cells are more suitable than the round ones used, for example, by bumblebees, as gaps are created between round cells and space is wasted. Square or triangular cells are another option, which are adjacent to each other and do not create unwanted gaps. However, the bee larvae that grow in the cells do not have any of the above-mentioned shapes in the cross-section, and this would again be a waste of space, in this case inside the cells.

**Figure 2:**
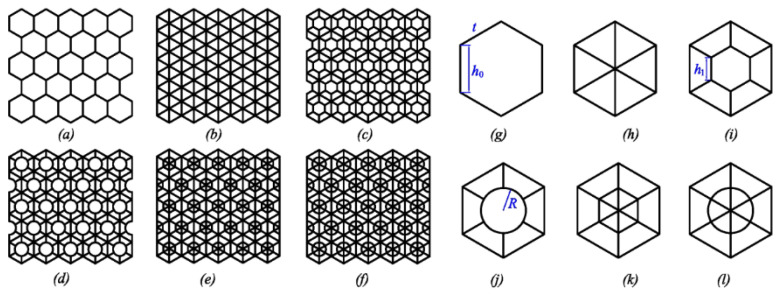
Honeycomb structures of cell interiors. (**a**) general structure; (**b**) triangular; (**c**) double hexagonal; (**d**) inside circular; (**e**) full double hexagonal; (**f**) full inside circular; (**g**) cell of hexagonal; (**h**) cell of full-triangular; (**i**) cell of double hexagonal; (**j**) cell of inside circular; (**k**) cell of full inside hexagonal; (**l**) cell of full inside circular [9].

Therefore, hexagonal cells are intuitively appropriate. Another remarkable strategy of bees, to save building material and space, is the two-layer hexagonal honeycomb. The bottom of each cell has a pyramid shape (again, a more efficient solution than a square bottom) and thanks to this, the opposite honeycombs fit perfectly [8]. Calculations and studies by many scientists have concluded that this structure is an almost optimal mathematical way to cover the maximum amount of space using the minimum amount of building materials [9,10]. In 1964, L.F. Tóth discovered the most economical ideal cell, which is hexagonal, but the bottom consists of two squares and two hexagons (see Figure 1) [1]. However, the saving was not that significant (less than 0.35%) compared to the complexity of the design. In an experiment with self-leveling soap bubbles [11], it was shown that, at a certain wall thickness, the ideal solution would shift from the optimal arrangement proposed by Toth to the arrangement preferred by the bees. Therefore, we can conclude that the structure is indeed very close to the theoretical one. Honeycomb structure with these kinds of cell interior have been presented in [9], see Figure 2.

## 2. Development of Artificial Honeycomb

Scientists, inspired by natural honeycombs, are trying to produce similar physical conformations in laboratories, which have been given the technical name of “honeycomb-like structures” (from the English abbreviation HCP). If we look at history and focus on the technological development of these porous structures, we can categorize this development into four periods [12]: (i) Period of first interest (60 BC-126): People were already fascinated by hexagonal honeycombs in antiquity. Several Greek mathematicians devoted themselves to the structure of the beehive, which is why the first results of their observations appear at this time [4,5]. This motif even appeared in the construction of the Pantheon in Rome, where the hexagonal structure formed the internal ribbing [13]. (ii) The period of observation and understanding (1638–1901): At first, the focus was on obtaining as much qualitative information as possible about the properties of beehives. This was also helped by the invention of the microscope in the 17th century, when, for example, the similarity between the cork structure and the honeycomb structure was observed [14]. With the advent of the industrial revolution, which connected science and technology, the demand for new lightweight materials grew, and the honeycomb structure became a hot topic. (iii) Period of application (1914–1990): It was not until the 20th century that the first attempts to artificially create a honeycomb-type structure with potential uses in many industries (aeronautics, cosmonautics, engineering, architecture, medicine [15,16]) appeared. In 1914, a structural application of honeycombs was patented [17], which was subsequently designed for construction applications. In the 1980s, the first HCPs were created from thermoplastics using the extrusion process [18]. (iv) The period of the interconnection of multiple fields and rapid development (1990-present): Due to the development and improvement of individual materials, technologies, applications, and the advent of nanotechnology, there was an exponential increase in publications on the topic of porous structures and honeycomb-like patterns [12]. At this time, the breath figure method was also discovered, thanks to which there was a rapid development of ordered HCPs from polymer materials.

## 3. Polymeric Honeycombs

Polymeric materials are suitable candidates for the preparation of microporous surface structures, as their properties can mimic those of natural honeycombs. These unique properties include a large specific surface area, high mechanical strength, good structural stability, thermal and sound insulation, and low density [19]. The relationships between the topology and mechanical, acoustic, and thermal properties of honeycomb structures are discussed by Zhang [12]. The unique properties of these structures mainly depend on the structure, the material used, the preparation process, and the final application. Depending on the polymers used, various aspects can be improved, by the introduction of additional functional groups or improvements in mechanical properties. While the physical aspects (thermal resistance, mechanical properties, etc.) are linked to the type of polymer that is used, the functionalization of HCP surfaces is undoubtedly a critical aspect of the usability of the structures [20].

**Figure 3:**
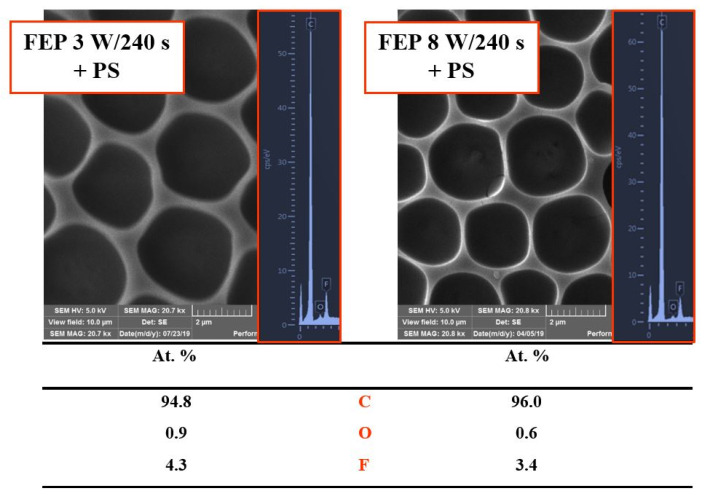
Scanning electron microscopy images (10 × 10 µm^2^) of plasma-modified fluorinated ethylene propylene (FEP) treated at different plasma discharges (3 and 8 W), with a polystyrene (PS) layer forming a honeycomb-like pattern. On the right side, corresponding energy-dispersive X-ray spectroscopy graphs and a table of element concentrations on the surface are depicted [27].

Standard commercial polymers were most often used for the preparation of porous structures due to their low price and availability; biopolymers may also be used [21]. These include, for example, polystyrene (PS) [22], cellulose acetate [23], polymethyl methacrylate (PMMA) [24], polylactic acid (PLA) [25], poly(e-caprolactone) (PCL). Furthermore, polymers with unique properties have been used, such as polymers that are responsive to external stimuli, biodegradable polymers, thermally and chemically stable polymers, and conductive and semi-conductive polymers. Furthermore, in addition to the type of polymer that is selected, macromolecular properties (molecular weight, polymer topology) play a key role in pore formation. Peng [26] observed the effect of molecular weight on the viscosity of the polymer solution and the subsequent surface morphology of the prepared porous membranes. When the molecular weight was low, the viscosity of the solution was too low to encapsulate the droplets needed to form ordered membranes or prevent them from coalescing. Conversely, a high molecular weight led to a highly viscous polymer solution and the droplets did not have time to wrap, resulting in gaps in the prepared membranes. The very first polymers used by the breath figure technique were star homopolymers. Thanks to the spherical shape that they form in solution, they can create highly ordered structures [27] (Figure 3). However, it was later shown that even linear homopolymers can form ordered porous films [28] and their use is undoubtedly the most widespread strategy. These include homopolymers, homopolymers with functional groups, and random and block copolymers. PS-based ones dominate the linear block copolymers because styrene is easily soluble in various solvents and is a standard precursor in the synthesis of block copolymers [3]. Other possible groups, according to topology, are grafted, branched and cyclic polymers. All these groups of polymers are suitable for the preparation of HCPs, and concrete examples were comprehensively described in the book by Rodríguez-Hernández [21]. Parameters such as average pore size or pore distribution depend to a large extent on the functional groups contained in the polymer chain. Polar groups are key in the breath figure technique because they stabilize the water droplets. For this reason, amphiphilic copolymers (polymers carrying hydrophilic groups) have been thoroughly studied for the preparation of HCP films, and proven to improve their construction [29]. The preferential orientation of hydrophilic groups to the pore wall occurs in the case of amphiphilic structures.

**Figure 4:**
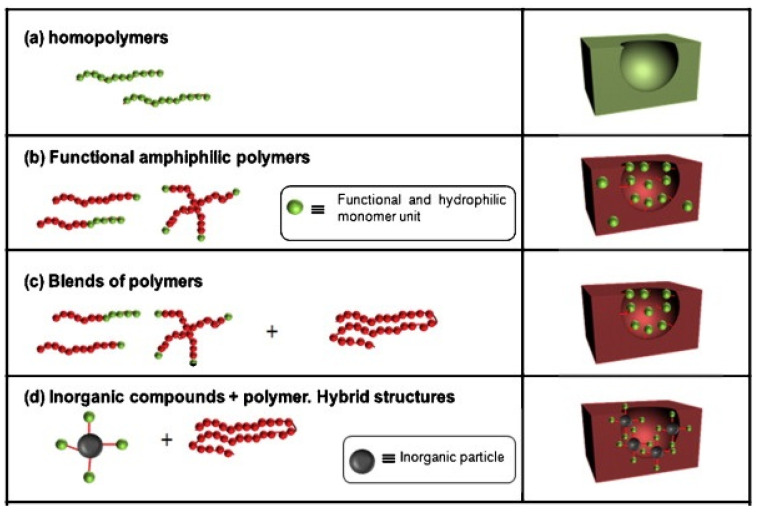
Various alternatives leading to functional surfaces being obtained in situ [2].

It is proven that even polymers without polar groups can form HCPs. For example, fluorinated polymers that have low surface free energy have also been used to produce porous films, especially superhydrophobic surfaces [30]. In addition to using a single polymer with appropriate functional groups, another alternative to the introduction of functionality and controlling hydrophobic/hydrophilic properties is the preparation of polymer blends. For example, blending homopolymers (such as PS) with amphiphilic copolymers significantly improves the regularity of porous-structured films. In Figure 4, we can see different approaches leading to functional micro-structured polymer films, as functionalization usually occurs in the pore wall [2]. The functionalization of porous films can also be achieved by introducing inorganic (metal nanoparticles, carbon nanotubes) or organic compounds [31]. Nanoparticles, such as metal/metal oxides, and quantum dots, are directly added to the polymer solution during the preparation of the HCP layers. During the condensation and growth process, small particles are adsorbed at the interface between the water droplets and the organic solvent. The adsorption of nanoparticles will not only help to stabilize water droplets but also provide new applications.

## 4. Preparation of HCP Structures

There are several methods to prepare honeycomb-like patterned surfaces. Both ordered and disordered structures can be prepared, depending on the method used and the applications. We can encounter many successful top-down approaches for the construction of ordered HCPs with controlled pore size and shape. These include, for example, various lithographic methods (photolithography, electron lithography, soft lithography), microprinting, pressing, and lasers [32,33]. Their disadvantages often include a high price, demanding and complex procedures, and can destroy and damage the prepared pattern. From an economic point of view, their surface instability is used for the construction of porous polymer films, which is based on bottom-up wetting techniques [34], phase separation [35], and “template methods” [36]. If the solvent starts to evaporate from the polymer solution, or if the undiluted mixture is heated above the glass transition temperature, phase separation will occur. The phase separation of polymer mixtures can lead to changes in the properties of the system (molecular weight, composition, structure, solvent, film thickness) or changes in the external environment (pressure, substrate, humidity, temperature) to different surface morphologies, and the formation of pores. An important parameter is the rate of solvent evaporation, which affects the size and symmetry of the formed pores. We can also include the breath figure method, which will be described in more detail in the next section. It is known that phase separation can be achieved by introducing a so-called “bad” solvent (e.g., methanol) into the polymer solution [37,38]. There is a local concentration of methanol and, thus, a phase rich in methanol is formed by the evaporation of the good solvent (e.g., chloroform). This phased process is responsible for the separation of the two phases. Water that condenses on the surface of the polymer film from the ambient air helps the growth of methanol droplets responsible for the formation of hexagonal pores [39]. When a poor solvent is used, in combination with a good solvent, it serves a dual role: it increases the number of nucleation sites and shortens the crystallization time of the polymer by increasing the extent of phase separation. The amount of methanol is expected to be responsible for the pore size. Two types of methods use templates: hard and soft [40]. The hard method uses monodisperse particles as a template, mostly colloidal silica particles or PS particles. The advantage of this method is simplicity and price, but it has significant drawbacks. The particles must be processed until they have a uniform size, and their removal is not easy often leading to structural defects in the porous materials. The soft template consists of block copolymers, the selected blocks of which are subsequently etched or dissolved. UV radiation, ozonolysis, chemical etching, and reactive ion etching are used for removal. This method can not only control the pore size but also realize the functionalization of ordered nano-porous polymer materials. However, there are still two disadvantages of a soft template: poor thermal and solvent resistance [41].

### 4.1. Breath Figure

A successful method based on phase separation is the “breath figure” (BF). The name of this method refers to the fact that dew occurs when breathing on a cold surface [42]. Since 1895, when J. Aitken investigated the formation of BF, this method was considered a natural phenomenon [43]. In his observations, he outlined that the resulting patterns were caused by a complex interplay between the physical and interphase processes acting on water vapor. Half a century later, dew formation, whose thermodynamic and kinetic aspects are key to understanding BF formations in polymeric materials, was studied theoretically and experimentally [44]. A breakthrough in the application of BF was achieved in 1994, when Francois et al., came up with the fabrication of polymer films with a highly ordered HCP morphology using condensed water droplets, which served as a dynamic template [27]. Star PS or polystyrene-b-polyparaphenylene dissolved in carbon sulfide was used in the experiment. The solution was applied to a solid substrate with a stream of moist air, and a regular pattern was formed after evaporation of the water and solvent. Since then, much attention has been paid to the preparation of HCP films with the help of BF. Many researchers, such as Stenzel, have carried out detailed research and extended the BF method to a wide range of polymers [28].

**Figure 5:**
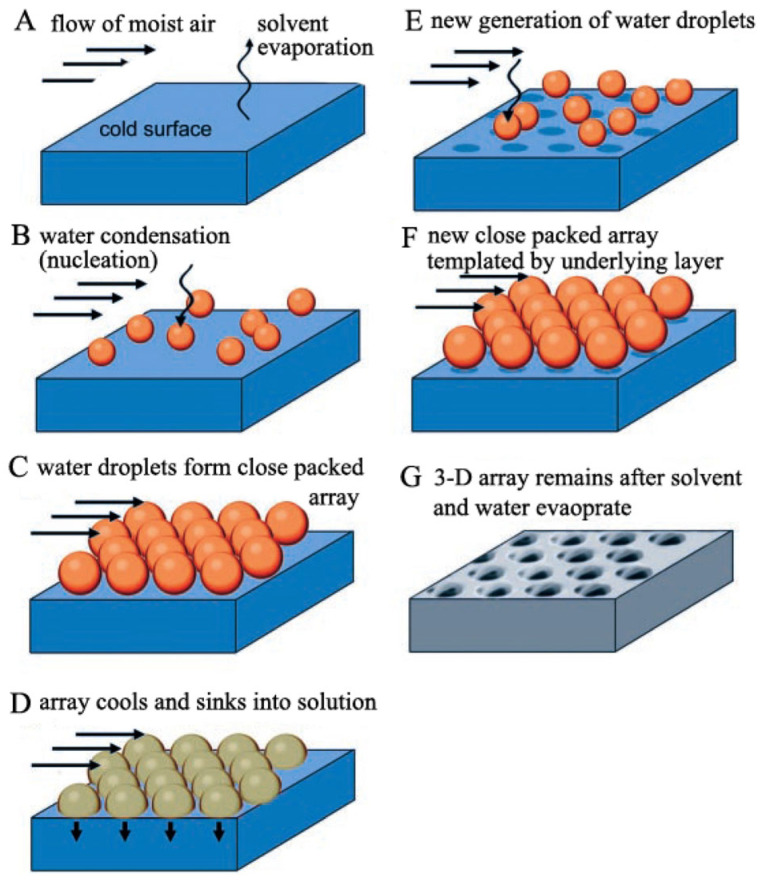
The BF method pore formation process [44].

The principle of the formation of porous membranes using the BF method is shown in Figure 5 [44]. As proposed by Munoz-Bonilla, the polymer is dissolved in a solvent, or mixture of solvents, and the solution is then applied to a substrate exposed to a stream of high-humidity air [2]. Evaporation of the volatile solvent lowers the surface temperature below the dew point, which leads to the rapid condensation and nucleation of water droplets on the surface of the polymer solution. Nucleation involves the formation of small droplets that do not evaporate because they are thermodynamically stable. During evaporation, the water droplets slowly grow at the expense of the vapor in the surrounding environment and, due to convection flow or capillary force, penetrating the surface of the solution, they pack into a hexagonal shape and serve as a template for the final HCP structure [45]. Once the surface temperature has equalized with the condensed water, the ambient temperature and remaining solvent evaporate to form the HCP. If the evaporation time is too short, the coalescence of water droplets occurs on the surface, and pores with random size and arrangement are formed. If the evaporation of the solvent ends before coalescence occurs, a homogeneous pore layer is formed. If the polymer stabilizes the condensed water droplets, and thus prevents precipitation, a situation may arise where the surface layer of condensed droplets sink into the solution. Whether a monolayer or a 3D porous network is formed depends on the density of the solvent used. When the solvent has a lower density than water, new droplets can condense and form another porous layer [2]. The key factors that determine the ordered structure in classical BFs are the stability of condensed water droplets and capillary force [46].

### 4.2. HCP Preparation Techniques Using the BF Method

Successful techniques for the preparation of porous structures using the BF method include spin-coating, drop-casting, and dip-coating [47]. The simplest and cheapest experimental technique using BF is drop-casting. The mixture of polymer and solvent is applied dropwise to the substrate. A relative humidity greater than 50% is usually required. Highly ordered structures are prepared by this technique. A modification of this basic technique has recently been developed to speed up the procedure by applying a series of drops in one step [48]. By applying an airflow with controlled humidity and flow, the rate of solvent evaporation can be controlled, and the flow indicates solution convection [49]. Compared to the direct application of the solution without airflow, there is a larger temperature gradient between the volume and the surface due to the faster evaporation. Control of the relative humidity, air flow rate, the concentration of dissolved polymer in solution or distance, and airflow angle are needed to optimize the resulting pattern. Films with lower roughness and homogeneous thickness can be prepared by the spin-coating method [50]. In this technique, the speed of rotation is an important factor. To ensure high ambient humidity during coating, containers filled with hot water are placed in the coating cell [51]. 

The dip-coating technique is also often used in combination with BF [52]. In this technique, the substrate is placed in a polymer solution in a vertical position. The speed of immersion and withdrawal from the solution plays a key role. Porous structures are mainly prepared on solid surfaces, including glass, silicon wafers, and polymers. An important feature of the carriers is their inertness towards solvents and suitable wettability. Plasma modification is an effective method, leading to an increase in the wettability of polymeric materials [53,54]. The action of the plasma discharge on the surface of the material results in the formation of reactive sites, cross-linking and changes in the chemical structure and molecular weight. This is a suitable strategy for incorporating functional groups, immobilizing proteins, and other biomolecules, or applying polymer coatings [55,56]. In addition, this treatment can be used to uniformly treat the surface of carriers of complex shape [57]. The air–water interface, the so-called “on-water spreading” approach, has been proven to be a suitable environment for the preparation of self-supporting structured films [58]. The morphology of the microstructure is controlled by the volume of the cast solution and the temperature of the water bath. Wan swapped water for ice and compared both interfaces, air–water, and air–ice. It was found that the polymer solution spread better at the air–ice interface [59]. To obtain highly ordered microporous films, the BF method requires the presence of specially structured polymers and appropriately designed devices that ensure precisely controlled high humidity [19]. These requirements limit the large-scale use of this method and its associated industrial applications. Farbod et al., used modified BF, known as “direct breath figure” (from the English direct breath figure, abbreviated DBF), to create a porous film. It is called direct because porous structures are created directly on the polymer substrate, and there is no need to apply an additional polymer film. It is, therefore, a top-down technique, as is the case with classic BF. The advantage of this method over classical BF is that DBF takes place without the presence of a moist surrounding atmosphere and the structure is a direct part of the substrate, retaining its mechanical stability [60]. However, the orderliness of the final pattern was not achieved, as is the case with classic BF. By combining the advantages of the BF and DBF techniques, a new breath figure method, called “semidirect breath figure” (sDBF), was created [61]. A pure solvent (in this case, chloroform) is applied to the substrate, replacing the polymer solution from classic BF. The method is as follows: (1) evaporation of chloroform in a humid environment and cooling of the substrate surface; (2) condensation of water; (3) formation of water droplets that serve as a template for the final hexagonal pattern; (4) swelling, dissolution and drying of the polymer surface; (5) evaporation of the remaining solvent and water and obtaining of a honeycomb structure, directly on the substrate.

### 4.3. Improved Phase Separation

**Figure 6:**
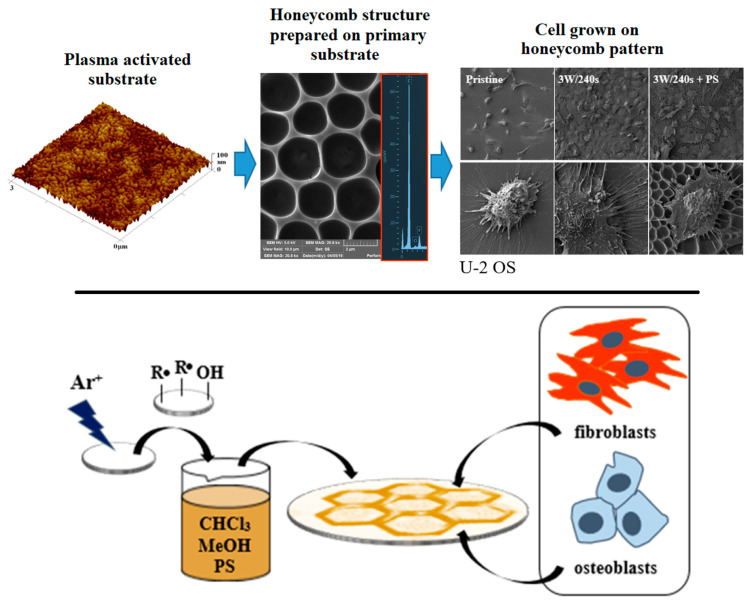
Scheme of the preparation of a polymer scaffold for cell culture [27].

Another suitable method for the preparation of HCPs is the “non-solvent-induced phase separation” (NIPS) method, which, unlike BF, enables the simple, large-scale production of porous patterns under the conditions of the surrounding atmosphere [38]. High humidity can be imitated by directly adding water or low-volatile solvents to polymer solutions [62], but the orderliness and homogeneity of the prepared films are not achieved. Bui prepared highly ordered honeycomb films from PLA without using high humidity [39]. This was achieved using a combination of the NIPS and BF methods, for which the name “improved phase separation” (from the English improved phase separation, abbreviated IPS) was adopted. This method has several advantages compared to the two mentioned above: it can be easily applied to industrial coating equipment, a large area can be covered, there is no need to control the air humidity or use surfactants, several cheap commercial polymers (PLA, PS) can be used (see Figure 6), and, in addition, the resulting morphology of the pattern can be controlled using the solvents and their ratio. 

This is a simple two-step method, where, in the first step, a polymer layer is applied to a solid substrate and, in the second step, the substrate is immersed in a mixture of two solvents, “good” (chloroform) and “bad” (methanol). After extracting the substrate from the binary mixture of solvents, they evaporate into the air. As chloroform is more volatile, it evaporates faster and methanol droplets accumulate on the surface of the polymer. At the same time, based on the BF principle [19,63], the rapid evaporation of chloroform cools the polymer surface, and high relative humidity is used to condense air water vapor into the polymer phase, which is rich in methanol. Water causes an acceleration in droplet growth and an increase in their surface tension. This is essential to the conformational stability of methanol droplets, when the droplets are tightly packed due to capillary forces and thus form a porous structure. The effect of humidity on the formation of HCPs was confirmed by Bui in an experiment in a dry environment [39], where no pattern was formed. Humidity does not have to be precisely regulated, but must be kept within a certain range (25–75%). This two-step preparation can limit the uniformity of the resulting HCP on complicated, uneven substrates. For this reason, Bui came up with a simplification of the existing IPS [52] and modified the two-step method to a one-step one without losing its advantages. 

The principle is the direct addition of methanol to the polymer dissolved in chloroform, and the formation of a ternary system: polymer–good-solvent–bad-solvent. Subsequently, the substrate is immersed in the prepared solution and an ordered porous structure is spontaneously formed by the evaporation of the solvents in the air. This approach can be applied to various complicated surfaces. The benefits of the suggested method depend on the relevant choice of methanol as a poor solvent, which induces phase separation under regular air and favors the creation of an ordered structure. Methanol, as a solvent, has three major impacts: (i) Methanol has a high affinity to water. Enriching the solution with methanol has a beneficial effect on the evaporation of solvents when the methanol absorbs water vapor from the surroundings. Accordingly, the phase separation that forms the resulting HCP can be induced in regular air. This preparation process has the potential to mass produce HCP films with high reproducibility and uniformity; (ii) Methanol’s high solubility in water-immiscible solvents, such as carbon disulfide, chloroform and dichloromethane, enables the creation of ordered porous structures [46]. Conversely, water-miscible solvents, such as tetrahydrofuran, are poor candidates for preparing an ordered structure. Thus, if a mixture of water and water-miscible solvents is used, the formation of an inhomogeneous structure is likely to occur; (iii) The presence of methanol causes the polymer’s transition to the surface of the poor solvent droplets. The formation of a gel-like layer occurs following the accumulation of polymeric material, which results in the droplet stabilizing against clotting. Therefore, a homogeneous structure can be achieved using commercial polymers. At some point, the local concentration of methanol induces a demixing process and forms two phases: (1) a polymer-rich chloroform phase containing a small amount of methanol, and (2) a methanol-rich phase containing a small amount of polymer and chloroform [64]. This phenomenon can be explained using the phase diagram of the thermodynamic behavior of the ternary-polymer–good-solvent–bad-solvent mixture [38].

## 5. Preparation Techniques and Their Variations

The most distinctive feature of HCP films is their unique morphology. The parameters of the morphology of porous polymer structures play an important role in the field of bioapplications. Specifically, in tissue engineering, the size and distribution of pores determines cells’ ability to attach, proliferate, and migrate across a substrate. Pore connectivity has been shown to affect cells’ ability to interact with each other, their migration rate, and the nutrient/waste diffusion if cell migration into the scaffold is required [65]. The surface morphology of the honeycomb films, including the size, shape, distribution, and density of the pores, is controlled by changing the conditions of their preparation. Controlling these parameters is necessary to achieve a highly ordered hexagonal structure. Environmental conditions (the humidity, airflow, temperature, and speed of preparation technique used), polymer solution concentration, solvent, and substrate are among the most important parameters to consider when preparing polymeric HCP structures [66]. Different pore shapes can also be created using mechanical deformation. Geometric patterns such as rectangles, elongated hexagons, triangles, and squares can be obtained by compressing or stretching the hexagonal micro-polymer films [67].

### 5.1. Modification of BF Method

A high ambient humidity is considered essential to the formation of a regular hexagonal pattern in the BF method. The pore size becomes larger with increased humidity in the surrounding environment. This is due to the higher number of water droplets that accumulate on the surface layer of the polymer solution. However, no pores will form on the film if the relative humidity of the atmosphere is too low [68]. Smaller pores are formed if a higher flow-rate of the surrounding air is reached, which evaporates the solvent faster. However, excessive air flow cannot provide the droplets with enough time to expand and arrange into a regular array [69]. 

**Figure 7:**
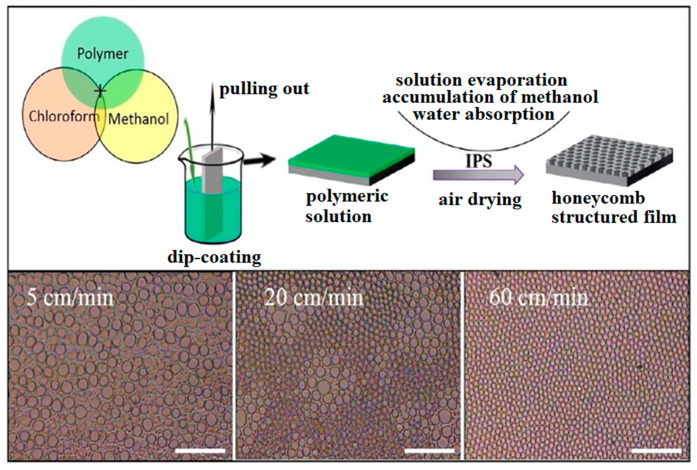
The effect that the speed of the substrate’s withdrawal from the solution using the dip-coating technique has on the morphology of the HCP layers [52].

Li et al., prepared HCP films with gold nanoparticles capped with dodecanethiol and controlled the velocity and direction of the moist air flow. The result was a change in the shape of the pores, from circles to ellipses [70]. A decrease in temperature caused an increase in the viscosity of the solution and its condensation and, at the same time, decreased the rate of solvent evaporation. As a result, larger pores and highly ordered HCP structures were formed [71]. Various BF preparation techniques were mentioned in the previous section. It is important to consider their speed when applying the solution. It has been shown that a higher spinning rate leads to a more homogeneous porous structure, and a lower spinning rate accompanied by slower evaporation leads to higher droplet coalescence. Furthermore, the pores become smaller as the rotation speed increases. This is because the water droplets have a shorter time to grow during faster evaporation. In general, the use of spin-coating techniques results in irregular patterns in comparison to the air-flow or drop-casting techniques. Moreover, pores created in this way tend to be less spherical and more elongated [72]. During the dip-coating technique, the withdrawal time from the polymer solution plays an important role. The faster the extraction from the solution, the smaller the resulting pores (see Figure 7) [52]. The pore dimension may enhance the cytocompatibility of the substrate [73], which will also be discussed in the next section. The viscous resistance of the solution increases as the draw-out speed increases. Thus, a higher pull-out speed leads to more solution on the sample. Moreover, to achieve phase separation and stabilization of the droplets, sufficient thickness of the solution layer is required. Thus, a higher pull-out speed leads to a higher homogeneity of the HCP layer [52]. A pressure drop can quicken the solvent evaporation, in the same way as the acceleration of air flowing over the surface of the solvent [74]. In the work of Kuroda et al., a way of creating arrangements of porous fields on the surface layer of the polymer using a vacuum is presented. The degree of vacuum is shown to affect the size of the pores that are formed [75].

### 5.2. Polymer

Additionally, the properties and type of polymer used affect the resulting morphology of the layer. For example, larger polymers are produced, with an increase in the polymeric molecular weight [76]. Deepak et al., showed the effect of polymeric substrate functionalization on pore formation [77]. In the case of a polymer mixture, the hydrophobic/hydrophilic component can dramatically affect the structure of the pore [50]. An important parameter controlling the morphology of the layer is the concentration of dissolved polymer in the solution. If the concentration of the polymer in the solution is large enough to keep the water droplets stabilized and prevent them from coalescing, then low concentrations usually lead to larger pores. If the polymer concentration in the solution is too high, unevenly distributed and shaped pores are formed. This is due to the high viscosity, which affects the arrangement of the water droplets forming the pores [78].

### 5.3. Solvent

The choice of solvent is a very important factor affecting the morphology of HCP films. Inherent solvent properties, such as volatility (the associated evaporation time) and solute solubility, surface tension between water and solution, and the thermodynamic affinity between the solute/solvent system and the boiling point of the solvent, govern the regularity and pore size. High volatility, and insolubility in water mean that water droplets do not mix with the polymer solution; a low boiling point, polymer precipitation, and good compatibility with the polymer are the basic requirements when choosing a solvent in the BF process [3]. High-volatility solvents usually lead to the creation of smaller pores due to the rapid evaporation of the solvent and the short time needed for water droplet growth [26]. During the evaporation of the solvent, the water droplet is stabilized by high interfacial tension. [79]. However, if the solvent has too high a boiling point, the solvent evaporates more slowly and evaporative cooling is not sufficient to condense water droplets from the atmosphere; for condensation to occur, there must be a difference in temperature between the surface of the solution and the dew point of the atmosphere. When using solvents with a lower boiling point, the difference between the surface temperature of the solution and the dew point increases, and more water condenses on the surface of the solution [80]. Methanol and chloroform, used as a system solvent–non-solvent, show very effective sensitivity for controlling pore size, distribution and shape. The average pore size considerably increases with an increase in non-solvent content in the mixture. This is due to the accumulation of more methanol in the non-solvent droplets and their enlargement [39]. When a poor solvent is used in combination with a good solvent, it plays a dual role: it increases the number of nucleation sites and shortens the crystallization time of the polymer by increasing the extent of phase separation [81]. Bui et al., used water-miscible and water-immiscible mixtures of solvents and non-solvents to create and govern the morphology on the surface of the resulting porous PMMA films and compared their effects [64].

### 5.4. Substrate

To prove the universality of the BF method, HCP layers have been formed on several solid [52] and liquid substrates [82]. The porous film is influenced by the substrate through bulk and surface properties, such as thermal conductivity, surface chemistry, and morphology. Furthermore, the size of the formed pores was shown to be related to the wettability of the substrate. While the hydrophobic samples resulted in the formation of smaller pores, the hydrophilic samples produced larger pores [83]. A higher pore arrangement was achieved on a thin glass substrate compared to a thick glass substrate. For this reason it is assumed that substrate thermal conductivity is involved in the film formation mechanism. It is important to use substrates that are not eroded by solvents, as organic solvents that are immiscible in water are mostly used. In most studies, authors use smooth substrates; however, they can also be prepared on various curved and structured surfaces, as the polymer solution copies the shape of the surface [84,85]. The total volume of condensed water droplets also increases by using a substrate with a higher surface roughness, which corresponds well with the increasing number of nucleation droplets [86].

## 6. Applications in Medicine

The possible uses of polymer HCP materials are very diverse. In biomedical uses, research is usually focused on increasing the efficiency and biocompatibility of these materials, which is an important aspect of tissue substitutes, scaffolds in tissue engineering [87,88,89], biosensors, gene carriers, and drugs [90,91]. Films with a honeycomb structure have chemical stability, good compatibility, controllable porosity, and flexibility in functionalization, which enables them to serve as excellent biological carriers. Furthermore, porous films are applied in the field of optical devices [92], ion separation [93], in the production of surface-enhanced Raman scattering (SERS) elements [94], superhydrophobic surfaces [95], and as electronic components [96].

### 6.1. Tissue Engineering

The field of tissue engineering mainly requires the regeneration or replacement of damaged tissue or organ. So-called “scaffolds” play a critical role in the development of new tissue in the interaction with human cells. These are material structures that have been designed to encourage cells to adhere, spread, migrate, proliferate and differentiate to form the desired tissue structure. Therefore, the chemical, mechanical and topological surface properties of scaffolds are important for regulating cell behavior [97]. The porous nature and unique surface topography of polymeric HCP layers make them suitable samples for investigating the various effects of cell topography behavior and for the production of biomimetic materials for use in tissue engineering [98]. The term “biomimetic polymers” refers to polymer-made materials that are focused on mimicking a structure, biological environment, or function to elicit the desired cellular responses. This definition refers to various design principles and materials from ligands interacting with receptors on the surface of cells to material structures with micro- and nanometer dimensions that cells can perceive and respond to in a specific way [99]. The many advantages that porous films provide include the increased permeability necessary for in vivo and in vitro tests, topographical stimuli influencing cell behavior, and the increased, spatially controlled adsorption of proteins. Another function of the pores can be as a protective and storage site for growth factors and bioadhesive molecules, or its use in the creation of site-specific immobilization sites for drug-delivery systems (DDS) [100]. 

The edges and pores themselves strengthen the cell’s ability to attach to the substrate during cultivation (increased cell adhesion) and can also mimic the essential microtopographic properties of the native extracellular matrix (ECM), further improving contact and interconnection in the horizontal direction and, thus, the possible formation of 3D structures. The pores’ presence throughout the structure can facilitate the flow of oxygen, nutrients, and waste products. An adequate flow of substances is an extremely important prerequisite for the survival of a cell and its function in cell culture media and transplantation [80,101]. Different pore sizes can serve as an effective tool to control cell growth or select different cell types [102]. 

**Figure 8:**
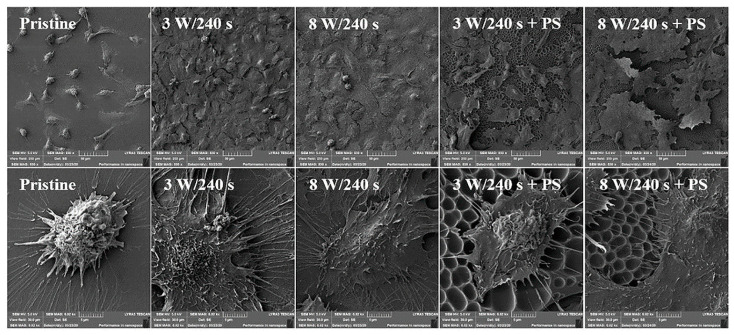
Scanning electron microscopy images of human osteoblasts (U-2 OS) six days post-seeding on FEP (pristine), FEP matrices treated by plasma with 3 W and 8 W (240 s) and subsequently coated with a honeycomb-like pattern formed from PS. The upper line represents a 250 × 250 µm^2^ scan, the bottom line a detailed scan with the area of 30 × 30 µm^2^. [27].

**Figure 9:**
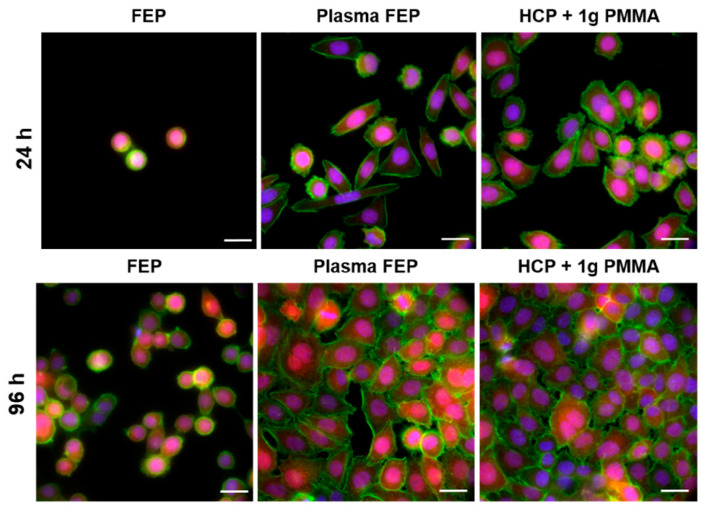
Fluorescence microscopy images of VAMPIRO U-2 OS cells (human cells derived from osteosarcoma, stably transfected with tFP602) growing on pristine fluorinated ethylene propylene (FEP) and FEP treated with 8 W plasma for 240 s and subsequently coated with a honeycomb-like pattern formed from polymethyl methacrylate (PMMA) solution (1 g), 24 h and 96 h post-seeding, respectively. Scale bar represents 20 microns [24].

An excellent review of studies dealing with polymeric porous structures as scaffolds in the field of tissue engineering has been presented by Calejo [98]. Biodegradable and amphiphilic polymers, including copolymers containing PCL, PLGA, PLA, PS, and others, were used for the preparation of scaffolds. Many types of eukaryotic cells were investigated, including stem cells, cancer cells, and normal cells, whose attachment to HCP films is shown in Figure 8 for polystyrene and U2-OS cells, while the fluorescent images of the same cell type on PMMA is displayed in Figure 9. It has been shown that HCP structures can support the adhesion of most normal cells, such as cardiac myocytes [102], endothelial cells [103], chondrocytes [104], osteoblasts [105], hepatocytes [72], fibroblasts and keratinocytes [106,107], NIH-3T3 cells [71], and some cancer cells, such as a cervical epithelial carcinoma cell line [108] and lung cancer cells [109]. Regular surface topography induced the differentiation of neural and mesenchymal stem cells [110,111]. Stem cells, as undifferentiated cells, have great potential in the field of tissue engineering, especially in cell therapy and disease modeling. This is due to their ability to differentiate into specialized cells in response to adequate signals and their high regenerative capacity. Surface topography can be applied in the field of cell culture to guide differentiation in the desired direction or maintain self-renewal without the use of any harmful chemicals [112].

### 6.2. Drug Delivery

Not only can structured films, thanks to their topography, control the “fate” of cells, they can also release therapeutics in a controlled manner into the surrounding medium (or into cells cultured on the material) or create reservoirs for various drug-delivery systems [113]. Due to its large specific surface area, the porous surface can accommodate a larger amount of therapeutics, even drugs with low solubility, which can be well dispersed in hydrophobic polymer matrices [114]. In addition, studies have shown that the encapsulation of drugs in porous layers contributes to a reduction in the so-called “burst release”, the negative effects of which are pharmacologically dangerous and economically inefficient [115]. Due to the highly porous nature of HCP films, the release characteristics of encapsulated pharmaceutical compounds may be significantly modified compared to non-porous films of the same material. It is, therefore, important to characterize such releases, especially as the polymer film degrades over time. Salicylic acid (highly soluble in water) and ibuprofen (slightly soluble in water) were selected as model drug compounds to characterize the release rate. The release rate of both compounds from a porous PLGA thin film was higher compared to an equivalent non-porous film [116]. 

Porous coatings with antifibrotic drugs, used for glaucoma drainage implants in the treatment of intraocular pressure, have shown very promising results [117,118]. The success rate of these uncoated implants is relatively low because the resulting fibrosis during the wound-healing process blocks fluid drainage. The porous coating prevents the formation of fibrosis, and thus reduces the occurrence of post-operative complications. Another study has shown the potential of double-layer porous PLGA platelets loaded with fenofibrate to successfully fight the growth of glioblastoma, a brain tumor. These platelets enabled a constant and steady release of the drug that actively inhibited the growth of tumor cells more effectively than a drug administered in a single dose or at high concentrations [119]. The wide distribution of pores and cavities can also serve as an ideal space for drug-delivery systems (DDS) [120]. For example, polymer microspheres [121] and nanoparticles [122] can be dispersed in a very simple way using gravity inside the pores of HCP films. Physical capture or electrostatic interaction can also be used. The advantage of the presence of secondary DDS lies in the greater load capacity of the system and the modulation of drug release, or in the possibility of achieving controlled drug release according to external stimuli such as temperature, light, ionic strength and pH [121,122]. Other research has shown that it is possible for porous polymer films whose cavities act as protein reservoirs to be used as a glucose-responsive insulin delivery tool, which may have potential as a controlled-release drug-delivery system for the treatment of diabetes [100].

### 6.3. Biosensors

Porous films are promising materials for the fabrication of highly sensitive biosensors. One example is a nanoelectrode formed by a polymer thin HCP film for the electrochemical detection of uric acid [123]. This biosensor can isolate the anodic peak in uric acid from that of ascorbic acid in cyclic voltammograms, which, without HCP layers, shows overlapping responses. The measurement of uric acid in human physiological fluids is used in the diagnosis and treatment of many renal and metabolic disorders, including renal failure, gout, leukemia, starvation, or other wasting conditions, and in the treatment of patients receiving cytotoxic drugs [124]. Chen reported the design of a patterned polymer film containing phenylboronic acid, exhibiting a high sensitivity for glucose detection [125]. The phenylboron part is hydrophilic and aggregates on the surface of water droplets, forming pore walls after evaporation. The BF method indicates the specific location of polar groups on the pore surface. Thanks to this, HCP films can sense biomolecules, such as proteins, through the presence of sugars on the surface of the pores [126,127]. Flexible touch sensors with high sensitivity are promising candidates for the production of artificial “electronic skin” for prosthetic devices or robotics. Such applications require inexpensive and scalable methods for producing pixels (units of sensing capacity) that sense medium and low pressures. For this reason, the BF method was chosen to create micro-structured and pattern-compressible dielectric layers for capacitive pressure sensors. The authors prepared porous polystyrene layers that served as molds for structuring polydimethylsiloxane dielectrics [128].

### 6.4. Antibacterial Layers

**Figure 10:**
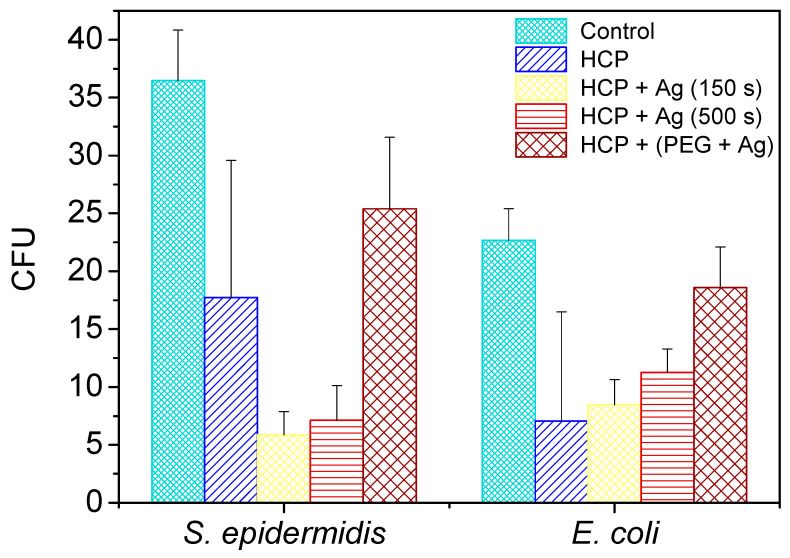
The number of colony-forming units (CFU) of *S. epidermidis* and *E. coli* were applied on the surface of the prepared samples of fluorinated ethylene propylene (FEP) for 2 h. The samples were tested after each phase of preparation: (1) A honeycomb-like (HCP) structure formation; (2) sputtering 150 s and 500 s of an Ag layer on the HCP surface; (3) sputtering of Ag into polyethylene glycol (PEG) and embedding the mixture into a solution of HCP structure. Bacterial suspension incubated only with phosphate-buffered saline without any sample addition served as a control. The samples were performed in triplicate [138].

Bacteria that adhere to the surface of materials, multiply and colonize to form biofilms often need to be controlled, especially on the surface of medical implants. The formation of a biofilm on an implant surface is one of the most common causes of failure [129]. The porous structure leads, in most cases, to an improvement in cell proliferation and may, therefore, raise concerns about increased bacterial colonization. It is essential to find effective strategies to prevent bacterial retention. Microporous surfaces have interesting properties that can enhance their antimicrobial effect [130]. This is very often achieved using antibacterial functional groups [131,132], organic and inorganic molecules [133], or different pore shapes and sizes. For example, films with pores with a diameter of 5–11 μm effectively prevent the attachment of *Pseudomonas aeruginosa* bacteria, a severe source of infection at present [134]. Vargas-Alfredo et al. [135] proposed the production of highly effective antibacterial substrates that are selective for bacteria, i.e., non-cytotoxic to mammalian cells. The proposed strategy is based on the different sizes of bacteria (1–4 μm) compared to mammalian cells (above 20 μm), which allow for the bacteria to contact the interior of the micrometer-sized pores containing antimicrobial functional groups. On the contrary, mammalian cells, which are larger, stay on the upper layer, thereby reducing unwanted cytotoxic effects and improving the biocompatibility of the substrates. Recently, Huang et al., used the same strategy and proposed a novel hybrid BF method for the preparation of functionalized HCP Petri dishes for cell culture [136]. The structure of mPEG, with its amphiphilic structure, activates OH groups that are located inside the pores and can covalently bind with other functional groups. Another effective strategy is doping polymer films with metal nanoparticles. One successful option is nanoparticles of silver (AgNPs), as they have good antifungal, antiviral and antibacterial properties (Figure 10) [137,138,139]. Ag, in its ionic form at low concentrations, acts as an antibacterial agent, although, for the Ag^0^ form, no significant antibacterial effect has been found. One example is an HCP film made of PLLA functionalized with Ag nanoparticles: the results show that the activity of Ag nanoparticles is more effective against *E. coli* bacteria in structured films compared to smooth films [140]. The addition of ferrocene to the PS solution showed an efficient way to control the Ag-functionalization and morphology of the surface of the fabricated HCP porous layers [141].

## 7. Modification of HCP Structures

In the previous section, the possible use of polymer layers with a porous structure in medicine was discussed. Modification by physical and chemical means, or a combination thereof, is an effective way to prepare HCP films with new properties. For example, in the field of tissue engineering, the most common goal is to create a surface that is attractive to cells, and increase their adhesion and proliferation on the surface of the carrier [142]. This can be achieved by optimizing the wettability and surface roughness, chemical composition, charge, and mechanical strength [143,144]. Some medical applications require antibacterial properties on the polymer surface, which can be prepared, for example, by adding metal nanoparticles or a PEG polymer [145]. The morphology of the surface and its nanostructuring are particularly important for the differentiation of stem cells and biosensors [146,147].

### 7.1. Physical Modification

**Figure 11:**
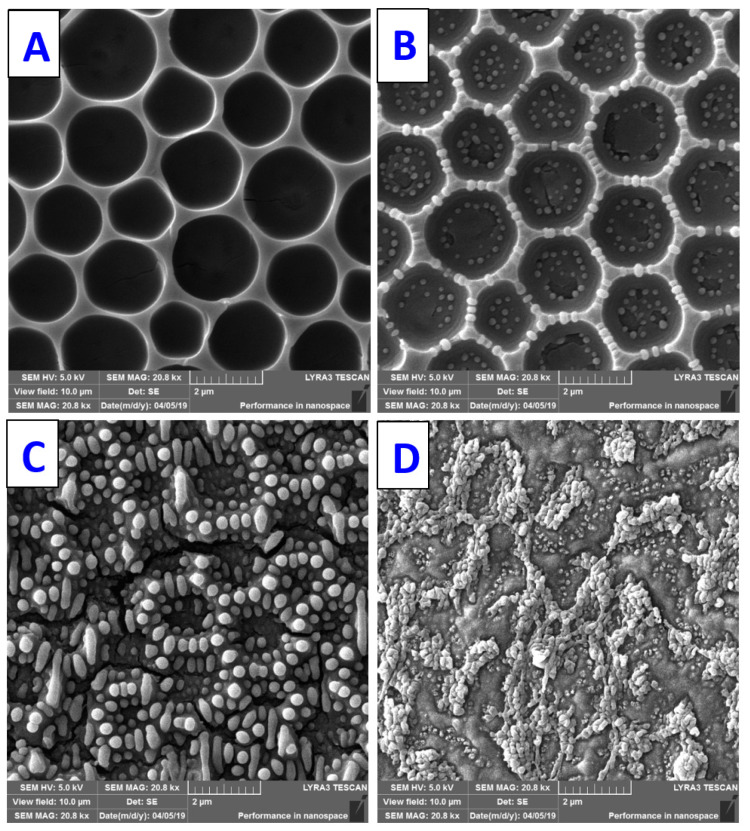
The different stages of modification of honeycomb-like PS patterns prepared on a FEP substrate plasma exposed to 8 W and 240 s (2 g of PS, ratio 90/10). The polystyrene HCP structures were exposed to an excimer laser with 6000 counts and laser fluence: (**A**)—0, (**B**)—6 (**C**)—10 and (**D**)—20 mJ.cm^−2^ [22].

Physical modifications are implemented on already-created HCPs and can be split into cross-linking and surface treatments [3]. The purpose of surface treatments can be, for example, the improvement in adhesion properties, change in hydrophobicity, the introduction of special functional groups to the surface, or the modification of surface morphology [148] (see Figure 11). 

One of the surface treatment methods that allows for the chemical and physical properties of polymer surfaces to be changed without affecting their bulk properties is treatment by plasma discharge [149]. Exposure of the polymer surface to plasma produces three main effects: surface ablation or etching, polymerization, and modification of the chemical structure of the exposed substrate. These phenomena lead to changes in wettability, surface energy, roughness, and chemistry, thereby contributing to the incorporation of functional groups or the attachment of proteins and cells to the polymeric material [150]. Yang et al., showed the incorporation of amino groups by plasma treatment, which promoted cell proliferation and attachment on a smooth PLA film [151]. In their next study, they combined the advantages of plasma treatment when incorporating amino functional groups and the excellent surface properties of HCP-patterned PLA to efficiently produce a biocompatible and cheap material [152]. Due to the presence of an external stimulus, such as a thermal or mechanical stress, osmotic pressure, or electric field, so-called “wrinkling instabilities” may appear, especially on the surface of polymers, which arise as a result of the system’s reaction to the loss of equilibrium. As a result, different surface morphologies can be observed with specific shapes and geometries at the nano- and micro-scale [153]. If the instabilities are created in a controlled manner, then periodically structured surfaces in polymeric material with modified properties of technological importance, such as adhesion, friction or wetting, can be obtained. Various laser techniques have been studied to create surface instabilities leading to the formation of surface topography. An example of laser technology is “laser-induced periodic surface structure” (LIPSS) [154]. Wrinkling is caused by repeated irradiation of the polymer surface with pulsed lasers. The probability of generating LIPSS on the polymer surface mainly depends on the polymer effectively absorbing the laser light [155]. Excimer laser exposure can, in special cases, lead to the formation of a globular structure on the surface of the polymer material [155]. 

Layers with the HCP pattern show hydrophobic behavior in most cases, due to their surface structure and the hydrophobic polymers from which they are prepared. Surface treatment of PS honeycomb films with UV-ozone caused the oxidation of surface hydrocarbons and thus increased surface wettability [156]. Another physical treatment can be the deposition of a thin organic (e.g., carbon) [157] or inorganic (e.g., metal, ceramic) [158,159] layer on the HCP surface. For example, a metal thin layer can be deposited on the substrate surface by sputtering in a vacuum [160]. In this way, a surface is created that has different chemical and structural properties to the original film. Not all foils produced by the BF method have good mechanical strength, which is desirable in often harsh environments. There is also a significant limitation in their use in some materials and their bioanalytical applications due to their solubility in organic solvents. Cross-linking is a commonly used strategy for modifying the obtained HCP films, especially when influencing their mechanical properties, and increasing thermal and chemical resistance. Cross-linking processes can be divided into three types: photochemical cross-linking, chemical cross-linking, and thermal cross-linking, all of which try to not damage the regularity of the films [3]. After chemical cross-linking, the films showed excellent resistance to high temperatures and various organic solvents and good mechanical strength [161]. Photochemical processing is another way to activate the cross-linking of porous films, principally when the casting solution contains double bonds. For example, in one study, films became chemically resistant to a variety of solvents and stable at 350 °C after 1 h of UV exposure. 

**Figure 12:**
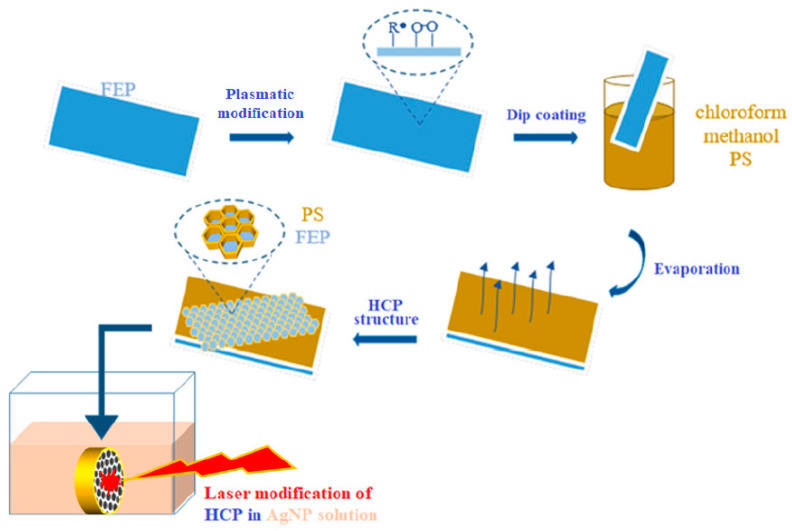
Scheme of the preparation of a honeycomb pattern immobilized with Ag nanoparticles. [164].

Additionally, photochemical cross-linking led to the formation of polar groups that converted the surface from hydrophobic to hydrophilic [109]. Microporous-structured thin films were prepared on functionalized PDLLA with photoreactive groups. Subsequent photo-crosslinking rendered the film insoluble in chloroform [162]. Thermal cross-linking is a one-step, effective method for treating polymeric HCP films. Through high-temperature processing, the groups with temperature-sensitive properties are cross-linked and part of the polymer microstructures collapse [3]. To preserve the microstructure, Bolognesi came up with a two-step heat treatment [163]. Physical modification can also include the targeted mechanical deformation of prepared HCP structures. Porous layers formed from elastic and viscoelastic polymers can expand or contract. The deformed shapes of the porous structures are characterized by the stretch/shrinkage ratio and the stretch/shrinkage direction [80]. The physical immobilization of noble metal nanoparticles from the solution can be simply realized by the exposure to an excimer laser in liquid (Figure 12), when the 248 nm wavelength is applied, a honeycomb composite is formed [164].

### 7.2. Chemical Modification

The in situ functionalization of HCP surfaces is often limited by polymers with an amphiphilic structure that facilitate the formation of a porous structure using the BF method. This section describes various chemical reactions and grafting methods that are used for ex situ modification of the chemical functionality of already-prepared porous layers [2]. These are substrate reactions with the solution when the chemical composition inside the pores changes by introducing a wide range of functional groups, or by linking organic or inorganic substances (molecules, nanoparticles, etc.). In particular, the immobilization of bioactive ligands has attracted intense interest due to the potential use of protein microarrays, which are not only suitable for the study of cell adhesion but also as biosensors and a material for tissue engineering [165,166]. Another challenge in microarray preparation is the site-specific immobilization of proteins to ensure that protein activity is not restricted [167]. Min et al., anchored biotin to the pore surface, which selectively interacts with the protein streptavidin [168]. A similar approach, using avidin–biotin non-covalent interactions, was described by Ke [169] and Nyström [170]. Linear polystyrene was mixed with amine-bonded polystyrene to prepare porous layers in which the functionality of the cavities could be modified. After the formation of BF, the hydrophilic amino groups were oriented toward the pore surface. Proteins were attached to the pore surface by cross-linking to amino groups using glutaraldehyde. The turning of hydrophilic amino groups towards the pore surface occurred after the formation of BF. On the surface of the pores, proteins were cross-linked to amino groups using glutaraldehyde [171]. The grafting of polymers is primarily an interesting method of chemical treatment of the material. The techniques of controlled radical polymerization provide a broad spectrum of polymers with narrow polydispersity on surfaces and controlled chain length using either a “grafting from” or “grafting onto” approach [2,172].

## 8. Conclusions

For the appropriate selection of the material and the effective use of its properties, it is necessary to study and understand the material’s surface and internal structure. We focused on the on formation of honeycomb microstructures and their applications, which include tissue engineering, antibacterial materials, replication processes or sensors. Thanks to this knowledge of the structure, it is possible to modify the existing properties of the material according to its intended application. Furthermore, surface nanostructuring provides a number of possibilities to modify or improve the properties of the studied material. We described experiments involving the breath figure process and the improved phase separation for honeycomb pattern formation, as well as subsequent surface modifications of the pattern.

## Data Availability

All data are contained within this article.
